# The Corona Immunitas Digital Follow-Up eCohort to Monitor Impacts of the SARS-CoV-2 Pandemic in Switzerland: Study Protocol and First Results

**DOI:** 10.3389/ijph.2022.1604506

**Published:** 2022-02-28

**Authors:** Alexandre Speierer, Patricia O. Chocano-Bedoya, Daniela Anker, Alexia Schmid, Dirk Keidel, Thomas Vermes, Medea Imboden, Sara Levati, Giovanni Franscella, Laurie Corna, Rebecca Amati, Erika Harju, Chantal Luedi, Gisela Michel, Caroline Veys-Takeuchi, Claire Zuppinger, Semira Gonseth Nusslé, Valérie D’Acremont, Ismaël Tall, Éric Salberg, Hélène Baysson, Elsa Lorthe, Francesco Pennacchio, Anja Frei, Marco Kaufmann, Marco Geigges, Erin Ashley West, Nathalie Schwab, Stéphane Cullati, Arnaud Chiolero, Christian Kahlert, Silvia Stringhini, Fabian Vollrath, Nicole Probst-Hensch, Nicolas Rodondi, Milo A. Puhan, Viktor von Wyl

**Affiliations:** ^1^ Institute of Primary Health Care (BIHAM), University of Bern, Bern, Switzerland; ^2^ Department of General Internal Medicine, Inselspital, Bern University Hospital, University of Bern, Bern, Switzerland; ^3^ Population Health Laboratory (#PopHealthLab), University of Fribourg, Fribourg, Switzerland; ^4^ Institute of Family Medicine, University of Fribourg, Fribourg, Switzerland; ^5^ Department of Epidemiology and Public Health, Swiss Tropical and Public Health Institute, Basel, Switzerland; ^6^ University of Basel, Basel, Switzerland; ^7^ Department of Business Economics, Health and Social Care, University of Applied Sciences and Arts of Southern Switzerland, Lugano, Switzerland; ^8^ Institute of Public Health, Faculty of BioMedicine, Università Della Svizzera Italiana, Lugano, Switzerland; ^9^ Department of Health Sciences and Medicine, University of Luzern, Luzern, Switzerland; ^10^ Department of Epidemiology and Health Systems, Center for Primary Care and Public Health (Unisanté), Lausanne University, Lausanne, Switzerland; ^11^ Department of Research and Innovation, Center for Primary Care and Public Health (Unisanté), Lausanne University, Lausanne, Switzerland; ^12^ Cantonal Public Health Service, Neuchâtel, Switzerland; ^13^ Department of Health and Community Medicine, Faculty of Medicine, University of Geneva, Geneva, Switzerland; ^14^ Unit of Population Epidemiology, Division of Primary Care Medicine, Geneva University Hospitals, Geneva, Switzerland; ^15^ Epidemiology, Biostatistics and Prevention Institute, Zurich, Switzerland; ^16^ Department of Readaptation and Geriatrics, University of Geneva, Geneva, Switzerland; ^17^ School of Population and Global Health, McGill University, Montreal, QC, Canada; ^18^ Department of Infectious Diseases and Hospital Epidemiology, Cantonal Hospital St. Gallen, St. Gallen, Switzerland; ^19^ Infectious Diseases and Hospital Epidemiology, Children’s Hospital of Eastern Switzerland, St. Gallen, Switzerland; ^20^ University Center for General Medicine and Public Health, University of Lausanne, Lausanne, Switzerland; ^21^ Corona Immunitas Program Management Group, Swiss School of Public Health, Zurich, Switzerland; ^22^ Institute for Implementation Science in Health Care, University of Zurich, Zurich, Switzerland

**Keywords:** prevention, digital follow-up, SARS-CoV-2, public health surveillance, population-based study, eCohort

## Abstract

**Objectives:** To describe the rationale, organization, and procedures of the Corona Immunitas Digital Follow-Up (CI-DFU) eCohort and to characterize participants at baseline.

**Methods:** Participants of Corona Immunitas, a population-based nationwide SARS-CoV-2 seroprevalence study in Switzerland, were invited to join the CI-DFU eCohort in 11 study centres. Weekly online questonnaires cover health status changes, prevention measures adherence, and social impacts. Monthly questionnaires cover additional prevention adherence, contact tracing apps use, vaccination and vaccine hesitancy, and socio-economic changes.

**Results:** We report data from the 5 centres that enrolled in the CI-DFU between June and October 2020 (covering Basel City/Land, Fribourg, Neuchâtel, Ticino, Zurich). As of February 2021, 4636 participants were enrolled and 85,693 weekly and 27,817 monthly questionnaires were collected. Design-based oversampling led to overrepresentation of individuals aged 65+ years. People with higher education and income were more likely to enroll and be retained.

**Conclusion:** Broad enrolment and robust retention of participants enables scientifically sound monitoring of pandemic impacts, prevention, and vaccination progress. The CI-DFU eCohort demonstrates proof-of-principle for large-scale, federated eCohort study designs based on jointly agreed principles and transparent governance.

## Introduction

Mitigating the consequences of the ongoing Severe Acute Respiratory Syndrome Coronavirus 2 (SARS-CoV-2) pandemic requires sound evidence on ever-changing medical, scientific, economic and social issues. Recent examples are the individual-level and population-based impacts of vaccinations [1, 2] or clinical and social implications of long COVID [3, 4].

Anticipating the need for a flexible study base, a consortium was founded in Switzerland in spring 2020. The Corona Immunitas research program has been described extensively elsewhere (www.corona-immunitas.ch) [5]. The primary aim of Corona Immunitas is to assess the nationwide, population-based seroprevalence (as measured by antibodies against nucleoid and spike proteins) of SARS-CoV-2 during different pandemic phases among randomly selected individuals from the general population, as well as in selected subgroups such as highly exposed healthcare workers or essential workers, such as bus drivers. To date, four waves of seroprevalence sampling (study phases covering different epidemiological situations) have been conducted. The first three study phases also saw an expansion of Corona Immunitas into further regions in Switzerland.

The Corona Immunitas Digital Follow-Up (CI-DFU) eCohort was planned as an integral part of Corona Immunitas from the outset, and all participants of the Corona Immunitas seroprevalence study were invited to join the CI-DFU. Whilst Corona Immunitas provides individual- and population-level assessments of natural and vaccine-induced immunity levels to SARS-CoV-2, the CI-DFU was designed to longitudinally follow Corona Immunitas seroprevalence study participants and to capture self-reported new and re-infections with SARS-CoV-2. Thereby, the CI-DFU contributes to the scientific evidence base on the extent and duration of antibody-mediated immunity. In addition, the CI-DFU eCohort aims to monitor physical and mental health effects of the pandemic, the adoption of preventive measures, changes in societal perception, socioeconomic changes and vaccine uptake.

Similar to the “parent” Corona Immunitas seroprevalence study, the CI-DFU is also regionally organized, thereby allowing study sites to address scientific questions of regional interests and fulfil local mandates of health authorities whilst contributing to the national monitoring via a standardized data collection protocol. This set-up has led to a decentralized organizational structure with agreed-upon data collection standards and questionnaires, which could potentially serve as a model for other longitudinal, regionally diverse research endeveaours.

The present article is structured as follows. The *Methods* section describes the rationale, organization, and procedures of the CI-DFU eCohort. In the *Results* section we present the characteristics of participants enrolled during CI study phase 2 (covering 6 cantons from all language regions) compared to the general population, thus evaluating representativeness of our sample. Further, we provide preliminary descriptions of factors associated with study follow-up retention. Finally, in the *Discussion* section, we present advantages and challenges of the decentralized CI-DFU organization structure, provide an overview of lessons learned and an outlook on upcoming topics to be addressed by the CI-DFU.

## Methods

### Rationale and Goals of the CI-DFU eCohort

The CI-DFU addressed the following research questions by means of weekly and monthly electronic questionnaires:

1) In individuals with prior exposure to SARS-CoV-2 (as confirmed by baseline serology), how long does the acquired immunity last and how many will experience a reinfection with SARS-CoV-2 based on self-reported symptoms and on self-reported PCR test results?
2) In individuals whose baseline serology was negative, how many will experience a self-reported infection with SARS-CoV-2?
3) How do individuals in Switzerland adjust their lives and adopt public health preventive measures to avoid contact with SARS-CoV-2 over extended periods of time?
4) How is mental health affected by the threat of the SARS-CoV-2 pandemic?

### Timeline of Study Roll-Out

The CI-DFU draws on the study base of the parent Corona Immunitas study. As described elsewhere [6], the parent Corona Immunitas seroprevalence study was rolled out in different phases. The Corona Immunitas study phase 1 (during the first lockdown in spring 2020) was initiated in Geneva [7]. Study phase 2 was launched in June 2020 and additionally included participants from the cantons of Basel (including Basel-City and Basel-Land), Fribourg, Neuchâtel, Ticino, Vaud and Zurich. Phase 3, launched in December 2020, saw the addition of participants from the cantons of Berne, Grison, Lucerne and St. Gallen.

**FIGURE 1:**
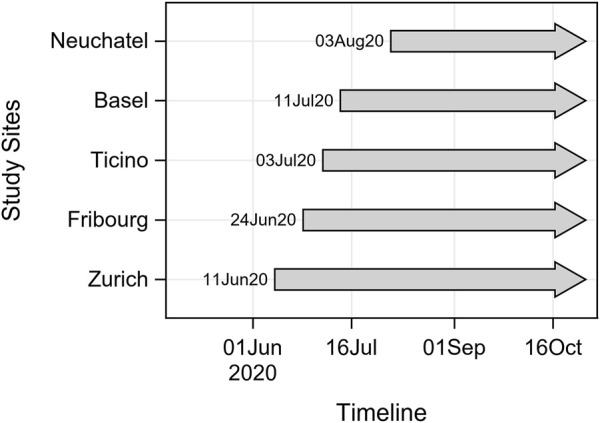
Timeline of enrolment during phase 2 (June 2020–October 2020) Corona Immunitas, Switzerland 2020.

Site-specific lauches of the CI-DFU were dependent on the roll-out schedule of the parent Corona Immunitas study, as well as on logistical and technical considerations (Figure 1). The following sites launched the digital follow-up during CI study phase 2 (starting in June/July 2021, henceforth referred to as “CI study phase”): Basel, Fribourg, Neuchâtel, Ticino and Zurich. The following sites launched the digital follow-up in late CI study phase 2 (November 2020) and CI study phase 3 (December 2020): Berne, Geneva, Grison, Lucerne, Vaud and St. Gallen.

### Study Participant Enrolment and Participation

The starting point for the CI-DFU of Corona Immunitas study was the on-site or at-home baseline visit at one of the 11 regional study centres (covering 12 cantons). Participants of the Corona Immunitas study have been randomly selected from the residential registry of participating cantons by the Swiss Federal Statistical Office, which excluded diplomats, persons with a foreign address in the registry, persons in asylum procedure, persons with a shortterm residence permit, and elderly people in nursing homes. Participants in Geneva phase 1 have been selected from the transversal population-representative study Bus Santé [8]. Enrolment into the CI-DFU eCohort was open to all participants of Corona Immunitas, given that they were at least 20 years of age (and younger age at some sites, Supplementary Tables S1, S2), provided informed consent in writing or online, had an email address and internet access. Trained study personnel were available to answer questions. While the invitations to join Corona Immunitas were sent only in the national languages (French, Italian and German) corresponding to each site, the informed consent forms and DFU questionnaires were available in four languages (with the addition of English). All study centers had a phone number in case participants had additional questions and were able to reply in the local languages. In addition, participants could use the contact form in the website in German, French, Italian or English (https://www.corona-immunitas.ch/en/contact-form/).

Enrolment procedures varied across sites (Supplementary Table S1). In most sites, participants were invited to the CI-DFU and provided informed consent during the initial on-site study visit for SARS-CoV-2 antibody-testing blood draw. In two sites (Basel and Ticino) however, participants started with the CI-DFU, and a sub-sample was invited to take part in the seroprevalence part of the parent CI-study. By design, all cantons except Ticino recruited 50% from the sample in the younger subgroup (20–64 years) and 50% in the older subgroup (65 years and older).

If participants consented to participate in the CI-DFU, they received questionnaire links *via* email on a weekly basis to provide electronic updates. The questionnaires were available in German, French, Italian and English. The repeated sequence were three short weekly questionnaires and a more extensive monthly questionnaire that could sometimes include secondary questionnaires on various topics. Initially, the CI-DFU was planned to run for up to 12 months after study enrolment. After all sites completed their recruitment goals, weekly participation rates ranged between 79 and 88% (Supplementary Figure S1). In light of the ongoing pandemic, participants who reached 12 months of follow-up were invited to extend study participation on an opt-in basis.

### Study Assessments

The variables to address the four main study questions were included in a standardized set of online core instruments. Through weekly online questionnaires participants in the CI-DFU reported on their health status and adherence to prevention measures for SARS CoV-2. Weekly questionnaires collected basic information concerning new occurrences of symptoms indicative of COVID-19, the new conduct of PCR- or rapid antigen SARS-CoV-2 tests and/or antibody serology. The results of these tests, hospitalizations, intensive care stays, and adherence to five recommended public health measures were also collected. The main goal of weekly status updates were to monitor possible (re-)infections with SARS-CoV-2 and the evolution of preventive behaviours in the face of a changing pandemic. The questionnaires were designed to take as little time as possible by implementing screening questions and branching logics. The time horizon of the questions refered to the past 7 days, that was, since the most recent questionnaire. Due to the high frequency of weekly questionnaires, invitations were only sent once without reminders.

Monthly, online questionnaires included additional topical issues such as the use of digital contact tracing apps, and SARS-CoV-2 vaccine hesitancy and vaccination status. The monthly questionnaires included additional instruments to assess further consequences of the pandemic, such as loneliness, social anxiety, psychological distress, or problems with access to healthcare and treatments. Finally, the questionnaires elicited participants’ perceptions of personal and societal risks concerning the SARS-CoV-2 pandemic and vaccinations. By default, invitations for participation were extended by email, and a reminder was sent in case the questionnaire had not been completed 4 days after invitation.

Whenever available, validated questionnaire instruments were utilized. Exact questions, wordings, and translations were reviewed by medical experts, data managers, and prospective participants and approved by all sites. The questionnaires were systematically reviewed on a regular basis and revised for clarity, alignment with new recommendations and medical developments (e.g., vaccines, new drugs, update of public health recommendations). Of note, the electronic CI-DFU questionnaires were synchronized with the parent Corona Immunitas study baseline questionnaire [extensively described elsewhere (5)]. Topic-specific questionnaires (e.g., on changes in socioeconomic status during the SARS-CoV-2 pandemic) were developed and added for specific projects. All DFU questionnaires are available as Supplementary Material (Annex 1).

While most sites followed a calendar-based sequence of CI-DFU questionnaires, that was, all participants received the same questionnaire on the same calendar date, one site had opted for a sequential study follow-up (Ticino). This means that participants all went through the same sequence of questionnaires at regular intervals, but the starting point (i.e., enrolment) determined the delivery date of a weekly or monthly questionnaire (Supplementary Table S1).

### Study Organization and Governance

In line with Corona Immunitas’ decentralized study design, individual study sites were responsible for implementing and delivering the CI-DFU questionnaires, although some sites opted for a centralized implementation of the online questionnaires by the University of Zurich (Berne, Fribourg, Grison, Luzern, Neuchâtel, St. Gallen and Zurich). Each site was responsible for the completeness of the database and could request to add or delete specific questions/questionnaires. Furthermore, sites could add additional questionnaire instruments to the monthly questionnaire according to their monitoring needs and local mandates.

Development of CI-DFU study aims, protocol and instruments was initiated in April 2020 by the national CI-DFU working group. This working group included site representatives and members of the central management team and was responsible for defining and further developing core study instruments, deciding on revisions and new additions, and coordinated the implementation of the CI-DFU across sites. The working group also initiated research projects and designs and implemented project-specific add-on questionnaires.

These tasks were executed in close collaboration with the Corona Immunitas Executive Committee and local principle investigators, who provided critical ideas and inputs. The national Corona Immunitas Executive Committee was responsible for strategic and scientific decisions and coordination, as well as the central communication team of Corona Immunitas, which organized and coordinated outreach and public relations activities. The Corona Immunitas Executive Committee also reviewed and approved project proposals and data access requests, for which clear guidelines had been established.

### Technical Infrastructure, Privacy Protection, and Data Quality

Maintaining privacy, data security, and data quality were top priorities of Corona Immunitas. The majority of sites used the REDCap (*Research Electronic Data Capture*) data collection system (https://www.project-redcap.org/) to capture and manage CI-DFU questionnaire data. Geneva utilized a specifically designed CRM-based digital platform system (https://www.specchio-covid19.ch/). REDCap is a secure, web-based software platform designed to support data capture for research studies, providing audit trails for tracking data manipulation and export procedures. REDCap allows for the management and automatic scheduling of invitations to online questionnaires [9, 10]. The data were collected through the standard REDCap web application interface, which facilitated questionnaire completion on any internet-connected device with an internet browser. REDCap also facilitated the implementation of quality control measures already at data entry, such as range checks, mandatory fields, and branching logics. Codebooks (questionnaire templates and table structures) and data dictionaries were exchanged between sites to ensure compatibility.

To protect privacy, direct communication with participants occured *via* local sites, only, and the research data were primarily captured and stored in systems hosted by local University IT services. For data quality checks and to ensure compatibility across sites, pseudonomised core CI-DFU data were periodically shared with the central data management of Corona Immunitas, which standardized the data further and compiled pseudonymized data files for national monitoring and research projects. The central data management communicated with local data management teams to address data collection and questionnaire implementation issues.

### Measures to Maintain and Improve Retention

Given the high frequency of questionnaires and the extended follow-up period, additional measures were designed to maintain high retention of CI-DFU participants. A strategy of Corona Immunitas consisted of “giving participants something back” by providing regionally customized newsletters including local news and (language) region-specific findings. Information about the study and findings were regularly shared in easily accessible formats such as short videos on the Corona Immunitas YouTube channel (Science in a Minute by SSPH+ available in German, French and Italian, https://www.youtube.com/c/scienceinaminute/videos) and through regularly updated data visualizations (e.g., of adherence to preventive measures from the follow-up questionnaires, available in four languages, including English: https://www.corona-immunitas.ch/en/news/behavioural-graphs/) on the Corona Immunitas website. These measures included also regular paper thank-you cards.

Participants could also provide direct feedback in the monthly questionnaire on their experiences while participating. These data not only provided valuable feedback but also material for testimonials and communication activities.

### Statistical Analysis

In this manuscript, we present key baseline characteristics of participants from the five Corona Immunitas sites that initiated digital follow-up assessments during CI study phase 2 in June 2020 (Basel, Fribourg, Neuchâtel, Ticino, Zurich). The analysis was restricted to individuals who were aged 20 years and older and who had completed at least one digital follow-up assessment. Baseline and follow-up information collected as of 2 February 2021 are shown. We describe the population of the study, as of 1 February 2021, with total numbers and percentages for categorical variables and median and interquartile ranges for continuous variables (age, body mass index). To report the follow-up percentage and retention, we divided the number of individuals with at least one completed follow-up questionnaire in February 2021 (that is, 3–6 months after enrolment) by the cumulative number of individuals with completed baseline assessments who agreed to take part in the Corona Immunitas CI-DFU and completed at least one digital survey. In addition, we used logistic regression to estimate the odds and 95% confidence intervals of having provided at least one (monthly or weekly) assessment in February 2021 to the CI-DFU (that is, 4–8 months after the launch of the CI-DFU eCohort).

### Ethics

The Corona Immunitas CI-DFU has been approved by the responsible ethics committees (Zurich: BASEC No 2020-01247, Geneva: BASEC No 2020-00881, Vaud: BASEC No 2020-00887, Basel: BASEC No 2020-00927, Ticino: BASEC No 2020-01514). All study participants have provided informed consent, which—depending on the site—was collected in written or online.

## Results

As of February 2021, the five analyzed CI-DFU eCohort regions with the longest follow-up (Basel, Fribourg, Neuchâtel, Ticino, Zurich) included 4636 participants with enrolment during CI study phase 2. Between June 2020 and February 2021, those participants completed 85,693 weekly and 27,817 monthly questionnaires.

### Participant Characteristics of Sites With Digital Follow-Up Launch in CI Study Phase 2 (Started June 2020)

The baseline characteristics of individuals from the five initial CI-DFU sites (Basel, Fribourg, Neuchâtel, Ticino and Zurich) with at least one complete CI-DFU assessment are shown in Table 1. Median age varied between 55 and 58 years in the four sites that also enrolled participants aged 65 years and older, while in Ticino, the median age was 46 years old because initial enrolment focused on individuals younger than 65 years. Between 40.0% and 52.6% of participants reported at least one of seven self-reported conditions that the Federal Office of Public Health considers as “high risk” (respiratory illness, immunocompromised, hypertension, cancer, cardiovascular disease, diabetes or allergies) [11]. Compared with official cantonal population statistics (Table 2), the age group of 65 years and older was overrepresented by design in those four sites, ranging from 30.5% to 42.8% of the adult population (compared with 20.6%–28.0% in the official population statistics). Furthermore, women were slightly over-represented among study participants at all sites, ranging from 51.1% in Zurich to 57.7% in Ticino. By comparison, official population statistics estimate the percentage of women to be between 50 and 51% (Table 2). Additionally, the proportion of participants without Swiss citizenship was lower in the eCohort (ranging from 7.4% in Fribourg to 17.3% in Ticino), when compared with official population statistics (ranging 22.8% in Fribourg to 28.6% in Basel).

**TABLE 1:** Baseline characteristics of the population of Basel (City/Land), Fribourg, Neuchâtel, Ticino, Zurich at time of enrolment during CI study phase 2 (June/July 2020) Corona Immunitas, Switzerland 2020.

	Basel (City/Land)	Fribourg	Neuchâtel	Ticino	Zurich
N	2269	308	284	978	797
Median age [interquartile range]	57.0 [46.0; 66.0]	55.0 [40.0; 67.0]	57.0 [43.0; 69.0]	46.0 [36.0; 55.0]	58.0 [44.0; 70.0]
Age categories					
20–64 years	1600, 70.5%	206, 66.9%	173, 60.9%	977, 99.9%	456, 57.2%
65+ years	669, 29.5%	102, 33.1%	111, 39.1%	1, 0.1%	341, 42.8%
Sex					
Female	1213, 53.5%	178, 58.0%	152, 53.5%	560, 57.4%	406, 50.9%
Male	1056, 46.5%	129, 42.0%	132, 46.5%	413, 42.4%	391, 49.1%
Other/Missing	0, 0.0%	1, 0.3%	0, 0.0%	5, 0.5%	0, 0.0%
Citizenship					
Swiss	1690, 74.5%	258, 83.8%	206, 72.5%	671, 68.6%	580, 72.8%
Swiss and other (dual citizenship)	244, 10.8%	25, 8.1%	42, 14.8%	139, 14.2%	91, 11.4%
Other	651, 14.0%	64, 7.4%	77, 11.3%	172, 17.3%	126, 15.8%
Highest school certificate					
No school certificate or mandatory school	52, 2.3%	15, 4.9%	18, 6.3%	31, 3.2%	26, 3.3%
Professional training	725, 32.0%	121, 39.3%	93, 32.7%	342, 35.0%	304, 38.1%
Matura, baccalaureate, vocational baccalaureate	148, 6.5%	34, 11.0%	43, 15.1%	191, 19.5%	57, 7.2%
Higher technical college or university of applied sciences	548, 24.2%	62, 20.1%	56, 19.7%	138, 14.1%	198, 24.8%
University	790, 34.8%	75, 24.4%	73, 25.7%	264, 27.0%	203, 25.5%
Missing	6, 0.3%	1, 0.3%	1, 0.4%	12, 1.2%	9, 1.1%
Number of other people currently living in the same household					
0	424, 18.8%	50, 16.7%	49, 17.3%	143, 14.8%	141, 17.8%
1	1025, 45.4%	123, 41.0%	126, 44.4%	290, 30.1%	397, 50.0%
2	357, 15.8%	50, 16.7%	40, 14.1%	215, 22.3%	114, 14.4%
3	328, 14.5%	54, 18.0%	51, 18.0%	241, 25.0%	100, 12.6%
4	96, 4.2%	18, 6.0%	10, 3.5%	60, 6.2%	31, 3.9%
5 or more	29, 1.3%	5, 1.7%	8, 2.8%	15, 1.6%	11, 1.4%
Has at least one child aged 18 or younger at home	564, 24.9%	111, 36.0%	90, 31.7%	388, 39.7%	179, 22.5%
Current monthly (gross) household income (CHF)					
0–3000	186, 8.2%	17, 5.5%	26, 9.2%	40, 4.1%	70, 8.8%
3001–6000	504, 22.2%	77, 25.0%	67, 23.6%	195, 19.9%	209, 26.2%
6001–9000	582, 25.7%	85, 27.6%	74, 26.1%	238, 24.3%	204, 25.6%
9001–12000	420, 18.5%	57, 18.5%	55, 19.4%	141, 14.4%	106, 13.3%
12001–15000	205, 9.0%	25, 8.1%	16, 5.6%	81, 8.3%	66, 8.3%
15001–18000	123, 5.4%	16, 5.2%	20, 7.0%	33, 3.4%	34, 4.3%
18001–21000	48, 2.1%	12, 3.9%	9, 3.2%	15, 1.5%	9, 1.1%
21001 +	105, 4.6%	6, 1.9%	7, 2.5%	46, 4.7%	41, 5.1%
No answer	96, 4.2%	13, 4.2%	10, 3.5%	189, 19.3%	58, 7.3%
Work situation					
Working (part- or full-time)	1392, 61.3%	179, 58.1%	146, 51.4%	726, 74.2%	434, 54.5%
Retired	730, 32.2%	103, 33.4%	116, 40.8%	40, 4.1%	325, 40.8%
Experienced a change in professional worklife due to COVID-19					
Lost a job due to COVID-19	24, 1.1%	4, 1.3%	4, 1.4%	13, 1.3%	3, 0.4%
Working from home (home office) among those working part- or full-time	607, 43.6%	79, 44.1%	53, 36.3%	274, 37.7%	212, 48.8%
Median Body Mass Index [Interquartile range]	24.3 [21.9; 27.2]	24.5 [22.3; 27.4]	25.1 [22.3; 28.3]	24.2 [21.3; 27.4]	24.6 [22.3; 27.7]
Smoking status					
Smoking daily	227, 10.0%	30, 9.7%	32, 11.3%	189, 19.3%	87, 10.9%
Smoking occasionally	134, 5.9%	20, 6.5%	7, 2.5%	53, 5.4%	44, 5.5%
Former smoker	606, 26.7%	95, 30.8%	88, 31.0%	194, 19.8%	226, 28.4%
Never smoked	1302, 57.4%	163, 52.9%	157, 55.3%	542, 55.4%	440, 55.2%
Self-reported chronic disease associated with an elevated risk for severe SARS-CoV-2 disease course	1121, 49.4%	132, 42.9%	136, 47.9%	389, 39.8%	420, 52.7%
Respiratory illness	143, 6.3%	17, 5.5%	26, 9.2%	51, 5.2%	56, 7.0%
Immunocompromised	91, 4.0%	17, 5.5%	17, 6.0%	39, 4.0%	32, 4.0%
Hypertension	449, 19.8%	60, 19.5%	48, 16.9%	73, 7.5%	184, 23.1%
Cancer	69, 3.0%	10, 3.2%	8, 2.8%	11, 1.1%	26, 3.3%
Cardiovascular disease	153, 6.7%	22, 7.1%	18, 6.3%	26, 2.7%	74, 9.3%
Diabetes	70, 3.1%	18, 5.8%	13, 4.6%	17, 1.7%	36, 4.5%
Allergies	617, 27.2%	57, 18.5%	71, 25.1%	278, 28.7%	208, 26.1%
Previous SARS-CoV-2 PCR test results					
Already has had a positive SARS-CoV-2 test result	11, 0.5%	1, 0.3%	0, 0.0%	10, 1.0%	0, 0.0%
Has been tested negative for SARS-CoV-2 in the past	266, 11.7%	24, 7.8%	23, 8.1%	62, 6.3%	56, 7.0%
No test done	1992, 87.8%	283, 91.9%	261, 91.9%	906, 92.6%	741, 93.0%

**TABLE 2:** Demographics of adult persons (aged 20 years and older) of cantons included in CI study phase 2 enrolment of the CI-DFU eCohort (Source: Federal Office of Statistics, Switzerland 2020).

	Switzerland (n)	%	Basel (City/Land) (n)	%	Fribourg (n)	%	Neuchâtel (n)	%	Ticino (n)	%	Zurich (n)	%
N overall	8′606′033		485′312		321′783		176′496		351′491		1′539′275	
20–64	5′283′035	76.7	292′925	74.0	198′907	79.4	105′527	75.6	207′648	72.0	972′784	78.8
65 and older	1′605′800	23.3	102′960	26.0	51′496	20.6	33′980	24.4	80′718	28.0	262′396	21.2
Men	4′268′863	49.6	236′887	48.8	161′153	50.1	86′495	49.0	171′141	48.7	766′679	49.8
Women	4′337′170	50.4	248′425	51.2	160′630	49.9	90′001	51.0	180′350	51.3	772′596	50.2
Swiss	6′430′658	74.7	346′700	71.4	248′327	77.2	131′913	74.7	254′633	72.4	1′122′495	72.9
Non-Swiss	2′175′375	25.3	138′612	28.6	73′456	22.8	44′583	25.3	96′858	27.6	416′780	27.1

### Participation

Figure 1 illustrates the starting date of the CI-DFU for different sites. During the observation period, participation in February 2021 ranged from 74.9% in Fribourg to 92.8% in Basel (Table 3). As of February 2021, the CI-DFU has obtained a median of 26 status assessments (questionnaires) from each of the 4636 participants.

**TABLE 3:** Factors associated with response to follow-up surveys^a^ three to 6 months after inclusion in the CI-DFU eCohort for sites with enrolment during CI study phase 2 (June to October 2020, total follow-up duration by February 2021 ranges between 5 and 8 months, depending on site). Corona Immunitas, Switzerland 2020.

	Missed/no assessment	Completed ≥1 assessment	Adjusted odds Ratio
	530 (11.4%)	4106 (88.6%)	[95% CI]
Canton			
Basel (City/Land)	163, 7.2%	2106, 92.8%	Ref.
Fribourg	77, 25.1%	231, 75.0%	0.21 [0.15; 0.29]
Neuchâtel	54, 19.0%	230, 81.0%	0.31 [0.21; 0.43]
Ticino	145, 14.9%	833, 85.2%	0.61 [0.47; 0.80]
Zurich	91, 11.4%	706, 88.6%	0.57 [0.43; 0.75]
Age categories			
20–64 years	458, 13.4%	2954, 86.6%	Ref.
65+ years	72, 5.9%	1152, 94.1%	2.73 [1.94; 3.84]
Sex			
Female	262, 10.4%	2247, 89.6%	Ref.
Male	268, 12.6%	1853, 87.4%	0.65 [0.54; 0.79]
Other/Missing (not included because of model convergence problems)	n.d.	n.d.	n.d.
Citizenship			
Swiss	346, 10.2%	3059, 89.8%	Ref.
Swiss and other	64, 11.9%	477, 88.2%	0.90 [0.67; 1.20]
Other	120, 17.4%	570, 82.6%	0.62 [0.48; 0.80]
Has at least one child aged 18 or younger at home	206, 15.5%	1126, 84.5%	0.73 [0.59; 0.90]
Highest school certificate			
No school certificate or mandatory school	35, 24.6%	107, 75.4%	Ref.
Professional training	163, 10.3%	1422, 89.7%	2.21 [1.41; 3.46]
Matura, baccalaureate, vocational baccalaureate	66, 14.0%	407, 86.0%	2.00 [1.22; 3.28]
Higher technical college or university of applied sciences	104, 10.4%	898, 89.6%	1.95 [1.21; 3.12]
University	157, 11.2%	1248, 88.8%	1.93 [1.22; 3.06]
Does not want to answer (Ticino only)	5, 17.2%	24, 82.8%	1.57 [0.53; 4.63]
Current monthly (gross) household income (CHF)			
0–3000	54, 15.9%	285, 84.1%	Ref.
3001–6000	128, 12.2%	924, 87.8%	1.34 [0.93; 1.94]
6001–9000	135, 11.5%	1048, 88.6%	1.53 [1.05; 2.22]
9001–12000	65, 8.3%	714, 91.7%	2.42 [1.59; 3.68]
12001–15000	38, 9.7%	355, 90.3%	2.14 [1.32; 3.47]
15001–18000	19, 8.4%	207, 91.6%	2.62 [1.45; 4.74]
18001–21000	15, 16.1%	78, 83.9%	1.56 [0.79; 3.07]
21001+	17, 8.3%	188, 91.7%	2.59 [1.41; 4.76]
does not want to answer (Ticino only)	59, 16.1%	307, 83.9%	1.18 [0.76; 1.83]
Work situation			
Working full- or part-time (Not working/retired as References)	356, 12.4%	2521, 87.6%	1.23 [0.96; 1.58]
Smoking status			
Smoking daily	93, 16.5%	472, 83.5%	Ref.
Smoking occasionally (i.e., not daily)	39, 15.2%	219, 84.9%	1.03 [0.67; 1.58]
Former smoker	113, 9.4%	1096, 90.7%	1.55 [1.14; 2.12]
Never smoked	285, 11.0%	2319, 89.1%	1.34 [1.02; 1.75]
Has at least one self-reported chronic disease (no chronic diseases as reference)	223, 10.2%	1975, 89.9%	1.10 [0.90; 1.33]

a Associations with response to at least one follow-up questionnaire in the month of February 2021.

In addition, we explored study subject characteristics associated with participation in at least one CI-DFU assessment by February 2021 (Table 3). Individuals aged 65 years and older (Odds Ratio (OR) 2.73, 95% CI:1.94, 3.84) were more likely to have participated, males (OR: 0.65, 95% CI: 0.54; 0.79 compared with females) and participants without a Swiss citizenship were less likely (OR: 0.62, 95% CI: 0.48, 0.80 compared to individuals with a Swiss citizenship) to have participated in February 2021. Having a University diploma was associated with a higher retention (OR: 1.93, 95% CI: 1.22, 3.06, reference no schooling or mandatory schooling). The same pattern was also seen with respect to monthly income. Individuals in higher household income brackets were more likely to participate in follow-up assessments (OR for monthly income above 21,000 vs up to 3,000 CHF: 2.59, 95% CI: 1.41, 4.76).

## Discussion

The CI-DFU eCohort is an integral part of Corona Immunitas, the nationwide Swiss seroprevalence study. It aims to collect and assess important data on social, economic, and health aspects of the Swiss population in a constantly evolving pandemic situation. The median of 26 status questionnaires from 4636 participants enrolled in CI study phase 2 (as of February 2021) makes the CI-DFU by far the largest longitudinal study on SARS-CoV-2 in the general population of Switzerland and provides information on SARS-CoV-2 infections, adherence to preventive public health measures, mental and physical health, healthcare utilization, and SARS-CoV-2 uptake in a well-documented study population.

Owing to the population-based, random sampling-based enrolment scheme of the Corona Immunitas study, non-Swiss citizens, who are usually less likely to participate in research, and individuals of lower educational or socioeconomic status are included. This is a key strength of Corona Immunitas and the CI-DFU. However, compared with official statistics, the overall population structure is skewed towards more women and an older median age. While the questionnaires were available in four languages, the invitations to participate in Corona Immunitas were sent only in German, French or Italian, which could have disencouraged participation of people who do not speak these languages. However, the three main languages, in which the Corona Immunitas surveys are delivered, represent the main spoken language of 92.9% of Swiss permanent residents; English is spoken as the main language by 5.7% of permanent residents (https://www.bfs.admin.ch/bfs/en/home/statistics/population/languages-religions/languages.html). The sampling scheme a-priori also excluded non-permanent residents of Switzerland. Furthermore, individuals who regularly completed the CI-DFU questionnaires tended to be more highly educated and report higher household incomes. This can partially be explained by the opt-in selection mechanism, which also exists in a setting with a random sample.

Therefore, our data provide unique possibilities for pandemic monitoring and help to garner insight into the lived experiences of Swiss residents during pandemic times. As such, the CI-DFU eCohort complements other ongoing, longitudinal research studies (COVID-19 Social Monitor https://covid19.ctu.unibe.ch/, CovidNorms, https://covid-norms.ch/en/) by covering a larger, more diverse study population and offering a detailed perspective on physical and mental health outcomes. The regularly updated (longitudinal) information on preventive behaviours, attitudes and risk perceptions of SARS-CoV-2 also complements mostly cross-sectional population questionnaires in Switzerland (e.g, SRF sotomo questionnaires [13], COVID-19 International Student Well-Being Study [14]).

In Europe, several population-based seroprevalence studies have been conducted to date [15], but few of them included a follow-up cohort [16–18]. For example, in Luxembourg, as part of a national survey (CON-VINCE study), 1862 asymptomatic adults were randomly selected [16] and completed questionnaires about psychological and behavioural factors every 2 weeks for the first 2 months since April 2020 and a final follow-up 1 year after participants’ inclusion in the study is planned. In Germany, the KoCo19 study included more than 5000 randomly selected participants between April and June 2020, who completed an online household questionnaire and an online personal questionnaire, a daily digital diary about symptoms, outings, use of public transportation, social contacts as well as additional questions about the psychosocial and economic situation [17, 19]. Compared to these studies, the CI-DFU eCohort is nationwide and has a comparatively large sample size. Therefore, it is not only well positioned to inform health policy and mitigation measures in Switzerland, but also to study topics of general scientific relevance and interest for an international community.

Key strengths of the CI-DFU eCohort include the availability of most questionnaires in four languages (German, French, Italian, English; not all questionnaire language versions are provided by all sites), the coverage of culturally different regions, the continuously high retention, the detailed characterization of study participants, but also the flexibility to respond to emerging topics concerning the SARS-COV-2 pandemic and to facilitate nested research studies. Furthermore, the Corona Immunitas project undertakes substantial efforts to inform study participants, using tools such as newsletters or short videos communicating findings in lay language.

The nationwide coverage of the eCohort is also a considerable strength because the SARS-CoV-2 pandemic exhibits regionally distinct epidemic patterns, both with respect to incidence and timing [12]. Furthermore, the harmonized and yet federal study structure ensured a standardized core dataset and enabled the sites to address questions of local relevance. This regionalization likely furthers the local acceptance and promotes identification with the study. Nevertheless, the highly decentralized study design also requires substantial coordination efforts. The study also benefits from the clearly defined governance structure, the inclusion of scientists with different expertise, a generally open, collaborative team spirit, and the broad involvement in decision making. This ensures that the general principles and core study requirements are broadly accepted and supported by the regional sites.

The CI-DFU eCohort also has limitations. The study participation in the main CI-DFU eCohort follow-up is restricted to individuals aged 20 years and older who are able to complete questionnaires online (some sites also include persons younger than 20 years and children). The data collected in the CI-DFU eCohort are based on self-reports and possibly subject to common measurement biases (e.g., over- or underreporting, social desirability bias, etc.). Despite being based on a randomly selected study population, there is substantial self-selection of study participants, both at time of study enrolment and during follow-up retention. In most sites, participants of the CI-DFU are a subsample of the parent Corona Immunitas study, whose participants were incentivized by learning about their SARS-CoV-2 serostatus. Furthermore, the CI-DFU eCohort may attract individuals who, in general, may be less skeptical about the severity of SARS-CoV-2 and the usefulness of mitigation measures, as well as better educated, computer-affine individuals. Furthermore, despite substantial efforts for standardization of core data, there is still local heterogeneity in how the questionnaires are implemented, driven by the need for adaptation to local circumstances and fulfilment of local mandates. It is noteworthy that we report here only baseline characteristics of the participants from the first five sites that started recruitment during summer 2020.

To summarize, the CI-DFU eCohort constitutes an important data source on the state of the pandemic in its own right. The potential insights to be gained from the CI-DFU transcend the pandemic-related aspects and also include many methodological aspects. As large-scale health studies will increasingly move online to interview study participants directly, the CI-DFU offers lessons in how to maintain a large study base over extended periods of time.
