# Supplementary material for: The Corona Immunitas Digital Follow-Up eCohort to Monitor Impacts of the SARS-CoV-2 Pandemic in Switzerland: Study Protocol and First Results
Source: Int J Public Health. 2022 Feb 28;67:1604506. doi: 10.3389/ijph.2022.1604506 (PMC8919370; doi:10.3389/ijph.2022.1604506)
Supplement: Supplementary file 1 [file DataSheet3.pdf]

CORONA IMMUNITAS RESEARCH GROUP

Emiliano Albanese, MD, PhD (Institute of Public Health (IPH), Università Della Svizzera Italiana, Lugano, Switzerland); **Rebecca Amati,** PhD (Institute of Public Health (IPH), Università Della Svizzera Italiana, Lugano, Switzerland) **Antonio Amendola**, Msc (Department of Business Economics, Health and Social Care (DEASS),, University of Applied Sciences & Arts of Southern Switzerland (SUPSI), Switzerland)); **Daniela Anker**, PhD (Population Health Laboratory (#PopHealthLab), University of Fribourg, Switzerland; Institute of Primary Health Care (BIHAM), University of Bern, Switzerland); **Anna Maria Annoni**, Msc (Institute of Public Health (IPH), Università Della Svizzera Italiana, Lugano, Switzerland); **Andrew Azman**, PhD (Unit of Population Epidemiology, Division of Primary Care Medicine, Geneva University Hospitals, Geneva, Switzerland; Department of Epidemiology, Johns Hopkins Bloomberg School of Public Health, Baltimore, MD, USA; Institute of Global Health, Faculty of Medicine, University of Geneva, Geneva, Switzerland); **Frank Bally**, MD (Institut central des hôpitaux, Hôpital du Valais, Sion, Switzerland); **Bettina Balmer** (Epidemiology, Biostatistics and Prevention Institute, University of Zurich, Zurich, Switzerland); **Hélène Baysson**, PhD (Department of Health and Community Medicine, Faculty of Medicine, University of Geneva, Geneva, Switzerland); **Delphine Berthod**, MD (Institut central des hôpitaux, Hôpital du Valais, Sion, Switzerland); **Kleona Bezani,** Msc (Institute of Public Health (IPH), Università Della Svizzera Italiana, Lugano, Switzerland); **Jacob Blankenberger** (Epidemiology, Biostatistics and Prevention Institute, University of Zurich, Zurich, Switzerland); **Murielle Bochud**, MD, PhD (Center for Primary Care and Public Health (Unisanté), University of Lausanne, Switzerland); **Patrick Bodenmann**, MD, Msc (Center for Primary Care and Public Health (Unisanté), University of Lausanne, Switzerland); **Matthias Bopp** (Epidemiology, Biostatistics and Prevention Institute, University of Zurich, Zurich, Switzerland); **Peter Buttaroni** (Institute of Public Health (IPH), Università Della Svizzera Italiana, Lugano, Switzerland); **Audrey Butty**, MD (Center for Primary Care and Public Health (Unisanté), University of Lausanne, Switzerland); **Anne Linda Camerini,** PhD (Institute of Public Health (IPH), Università Della Svizzera Italiana, Lugano, Switzerland); **Céline Cappeli** (Epidemiology, Biostatistics and Prevention Institute, University of Zurich, Zurich, Switzerland); **Cristian Carmelli**, PhD (Population Health Laboratory (#PopHealthLab), University of Fribourg, Switzerland); **Arnaud Chiolero**, MD, PhD (Population Health Laboratory (#PopHealthLab), University of Fribourg, Switzerland; Institute of Primary Health Care (BIHAM), University of Bern, Switzerland; Department of Epidemiology, Biostatistics and Occupational Health, McGill University, Montréal, Canada); **Patricia Orializ Chocano-Bedoya**, MD, PhD (Institute of Primary Health Care (BIHAM), University of Bern; Population Health Laboratory (#PopHealthLab), University of Fribourg, Switzerland); **Prune Collombet** (Unit of Population Epidemiology, Division of Primary Care Medicine, Geneva University Hospitals, Geneva, Switzerland); **Laurie Corna**, PhD (Department of Business Economics, Health and Social Care (DEASS),, University of Applied Sciences & Arts of Southern Switzerland (SUPSI), Switzerland); Swiss School of Public Health); **Jenny Crawford** (Epidemiology, Biostatistics and Prevention Institute, University of Zurich, Zurich, Switzerland); **Luca Crivelli**, PhD (Department of Business Economics, Health and Social Care (DEASS), University of Applied

Sciences & Arts of Southern Switzerland (SUPSI), Switzerland); Institute of Public Health (IPH), Università Della Svizzera Italiana, Lugano, Switzerland); **Stéphane Cullati,** PhD (Population Health Laboratory (#PopHealthLab), University of Fribourg, Switzerland; Department of Readaptation and Geriatrics, University of Geneva, Switzerland); **Valérie D'Acremont**, MD, PhD (Center for Primary Care and Public Health (Unisanté), University of Lausanne, Switzerland; Swiss Tropical and Public Health Institute, Basel, Switzerland); **Diana Sofia Da Costa Santos** (Institute of Public Health (IPH), Università Della Svizzera Italiana, Lugano, Switzerland); Agathe Deschamps (Cantonal Medical Service Neuchâtel); **Alexis Dumoulin**, PhD (Institut central des hôpitaux, Hôpital du Valais, Sion, Switzerland); Olivier Duperrex, MD,MSc (Center for Primary Care and Public Health (Unisanté), University of Lausanne, Switzerland); **Julien Dupraz**, MD, MAS (Center for Primary Care and Public Health (Unisanté), University of Lausanne, Switzerland); **Malik Egger** (Center for Primary Care and Public Health (Unisanté), University of Lausanne, Switzerland); **Nathalie Engler** (Cantonal Hospital St. Gallen, Clinic for Infectious Diseases and Hospital Epidemiology, St. Gallen, Switzerland); **Adina Mihaela Epure**, MD (Population Health Laboratory (#PopHealthLab), University of Fribourg, Switzerland; Department of Epidemiology and Health Services, Center for Primary Care and Public Health (UNISANTÉ), University of Lausanne, Lausanne, Switzerland); **Lukas Erksam** (Institute of Primary Health Care (BIHAM), University of Bern, Department of General Internal Medicine, Inselspital, Bern University Hospital, University of Bern); **Sandrine Estoppey** (Center for Primary Care and Public Health (Unisanté), University of Lausanne, Switzerland); **Marta Fadda**, PhD (Institute of Public Health (IPH), Università Della Svizzera Italiana, Lugano, Switzerland); **Vincent Faivre** (Center for Primary Care and Public Health (Unisanté), University of Lausanne, Switzerland); **Jan Fehr**, MD (Epidemiology, Biostatistics and Prevention Institute, University of Zurich, Zurich, Switzerland); **Andrea Felappi** (Center for Primary Care and Public Health (Unisanté), University of Lausanne, Switzerland); **Maddalena Fiordelli**, PhD (Institute of Public Health (IPH), Università Della Svizzera Italiana, Lugano, Switzerland); **Antoine Flahault**, MD, PhD (Institute of Global Health, Faculty of Medicine, University of Geneva, Geneva, Switzerland; Division of Tropical and Humanitarian Medicine, Geneva University Hospitals, Geneva, Switzerland; Department of Health and Community Medicine, Faculty of Medicine, University of Geneva, Geneva, Switzerland); **Luc Fornerod**, MAS (Observatoire valaisan de la santé (OVS), Sion, Switzerland); **Cristina Fragoso Corti**, PhD (Department of environment construction and design (DACD, University of Applied Sciences & Arts of Southern Switzerland (SUPSI), Switzerland); **Irène Frank**, PhD (Luzerner Kantonsspital, Spitalstrasse, 6000 Luzern 16); **Giovanni Franscella**, Msc (Institute of Public Health (IPH), Università Della Svizzera Italiana, Lugano, Switzerland**); Anja Frei**, PhD (Epidemiology, Biostatistics and Prevention Institute, University of Zurich, Zurich, Switzerland); **Marco Geigges**, PhD (Epidemiology, Biostatistics and Prevention Institute, University of Zurich, Zurich, Switzerland); **Doreen Gille** (Epidemiology, Biostatistics and Prevention Institute, University of Zurich, Zurich, Switzerland); **Semira Gonseth Nusslé**, MD, MSc (Center for Primary Care and Public Health (Unisanté), University of Lausanne, Switzerland); **Idris Guessous**, MD, PhD (Unit of Population Epidemiology, Division of Primary Care Medicine, Geneva University Hospitals, Geneva, Switzerland; Department of Health and Community Medicine, Faculty of Medicine, University of Geneva, Geneva,

Switzerland); **Huldrych Günthard** (Epidemiology, Biostatistics and Prevention Institute, University of Zurich, Zurich, Switzerland); **Felix Gutzwiller** (Epidemiology, Biostatistics and Prevention Institute, University of Zurich, Zurich, Switzerland); **Erika Harju**, PhD (Department of Health Sciences and Medicine, University of Lucerne, Frohburgstrasse 3, 6002 Lucerne); **Medea Imboden**, PhD (Swiss TPH, Basel, Switzerland; University of Basel, Basel, Switzerland); **Emilie Jendly** (Center for Primary Care and Public Health (Unisanté), University of Lausanne, Switzerland); **Ruedi Jung**, MSc (Epidemiology, Biostatistics and Prevention Institute, University of Zurich, Zurich, Switzerland); **Christian Kahlert**, MD (Cantonal Hospital St. Gallen, Clinic for Infectious Diseases and Hospital Epidemiology, St. Gallen, Switzerland; Children's Hospital of Eastern Switzerland, Infectious Diseases and Hospital Epidemiology, St. Gallen, Switzerland); **Laurent Kaiser**, MD, PhD (Geneva Center for Emerging Viral Diseases and Laboratory of Virology, Geneva University Hospitals, Geneva, Switzerland; Division of Infectious Diseases, Geneva University Hospitals, Geneva, Switzerland; Department of Medicine, Faculty of Medicine, University of Geneva, Geneva, Switzerland); **Laurent Kaufmann** (Service de La Santé Publique, Canton de Neuchâtel, Neuchâtel, Switzerland); **Marco Kaufmann** (Epidemiology, Biostatistics and Prevention Institute, University of Zurich, Zurich, Switzerland); **Simone Kessler** (Cantonal Hospital St. Gallen, Clinic for Infectious Diseases and Hospital Epidemiology, St. Gallen, Switzerland); **Philipp Kohler**, MD, MPH (Cantonal Hospital St. Gallen, Clinic for Infectious Diseases and Hospital Epidemiology, St. Gallen, Switzerland); **Christine Krähenbühl** (Luzerner Kantonsspital, Spitalstrasse, 6000 Luzern 16); Susi Kriemler, MD (Epidemiology, Biostatistics and Prevention Institute, University of Zurich, Zurich, Switzerland); **Julien Lamour** (Unit of Population Epidemiology, Division of Primary Care Medicine, Geneva University Hospitals, Geneva, Switzerland); **Sara Levati**, PhD (Department of Business Economics, Health and Social Care (DEASS), University of Applied Sciences & Arts of Southern Switzerland (SUPSI), Switzerland); **Elsa Lorthe,** RM, PhD (Unit of Population Epidemiology, Division of Primary Care Medicine, Geneva University Hospitals, Geneva, Switzerland); **Bettina Maeschli** (Epidemiology, Biostatistics and Prevention Institute, University of Zurich, Zurich, Switzerland); **Jean-Luc Magnin**, PhD (Laboratory, HFR-Fribourg, Fribourg, Switzerland); **Eric Masserey** (Cantonal Medical Office, General Health Department, Canton of Vaud, Switzerland); **Gisela Michel**, PhD (Department of Health Sciences and Medicine, University of Lucerne, Frohburgstrasse 3, 6002 Lucerne); **Rosalba Morese**, PhD (Institute of Public Health (IPH), Università Della Svizzera Italiana, Lugano, Switzerland); **Nicolai Mösli** (Swiss TPH, Basel, Switzerland; University of Basel, Basel, Swtizerland); **Natacha Noël** (Unit of Population Epidemiology, Division of Primary Care Medicine, Geneva University Hospitals, Geneva, Switzerland); **Daniel Henry Paris**, MD PhD (Swiss TPH, Basel, Switzerland; University of Basel, Basel, Swtizerland); **Jérôme Pasquier,** PhD (Center for Primary Care and Public Health (Unisanté), University of Lausanne, Switzerland); **Francesco Pennacchio**, PhD (Unit of Population Epidemiology, Division of Primary Care Medicine, Geneva University Hospitals, Geneva, Switzerland**); Stefan Pfister**, PhD (Laboratory, HFR-Fribourg, Fribourg, Switzerland); **Giovanni Piumatti**, PhD (Institute of Public Health (IPH), Università Della Svizzera Italiana, Lugano, Switzerland) **Nicole Probst-Hensch**, Dr. phil.II, PhD, MPH (Swiss TPH, Basel, Switzerland; University of Basel, Basel, Swtizerland); **Caroline Pugin** (Unit of Population

Epidemiology, Division of Primary Care Medicine, Geneva University Hospitals, Geneva, Switzerland); **Milo Puhan**, MD, PhD (Epidemiology, Biostatistics and Prevention Institute, University of Zurich, Zurich, Switzerland); **Nick Pullen,** PhD (Unit of Population Epidemiology, Division of Primary Care Medicine, Geneva University Hospitals, Geneva, Switzerland); **Thomas Radtke**, PhD (Epidemiology, Biostatistics and Prevention Institute, University of Zurich, Zurich, Switzerland); **Claude-François Robert** (Cantonal Medical Service Neuchâtel); **Pierre-Yves Rodondi**, MD (Institute of Family Medicine, University of Fribourg, Fribourg, Switzerland); **Nicolas Rodondi**, MD, MAS (Institute of Primary Health Care (BIHAM), University of Bern; Department of General Internal Medicine, Inselspital, Bern University Hospital, University of Bern); **Javier Sanchis Zozaya**, MD (Center for Primary Care and Public Health (Unisanté), University of Lausanne, Switzerland); **Virginie Schlüter**, MAS (Center for Primary Care and Public Health (Unisanté), University of Lausanne, Switzerland); **Alexia Schmid**, MSc (Institute of Family Medicine, University of Fribourg, Fribourg, Switzerland); **Valentine Schneider** (Cantonal Medical Service Neuchâtel); **Maria Schüpbach** (Institute of Primary Health Care (BIHAM), University of Bern, Department of General Internal Medicine, Inselspital, Bern University Hospital, University of Bern); **Nathalie Schwab** (Institute of Primary Health Care (BIHAM), University of Bern, Department of General Internal Medicine, Inselspital, Bern University Hospital, University of Bern); **Alexandre Speierer** (Institute of Primary Health Care (BIHAM), University of Bern; Department of General Internal Medicine, Inselspital, Bern University Hospital, University of Bern); **Amélie Steiner-Dubuis** (Center for Primary Care and Public Health (Unisanté), University of Lausanne, Switzerland); **Silvia Stringhini,** PhD (Unit of Population Epidemiology, Division of Primary Care Medicine, Geneva University Hospitals, Geneva, Switzerland; Department of Health and Community Medicine, Faculty of Medicine, University of Geneva, Geneva, Switzerland); **Johannes Sumer**, MD (Cantonal Hospital St. Gallen, Clinic for Infectious Diseases and Hospital Epidemiology, St. Gallen, Switzerland); **Julien Thabard** (Center for Primary Care and Public Health (Unisanté), University of Lausanne, Switzerland**); Nicolas Troillet**, MD, MSc (Institut central des hôpitaux, Hôpital du Valais, Sion, Switzerland); **Agne Ulyte**, MD (Epidemiology, Biostatistics and Prevention Institute, University of Zurich, Zurich, Switzerland); **Sophie Vassaux** (Center for Primary Care and Public Health (Unisanté), University of Lausanne, Switzerland); **Thomas Vermes**, MSc (Swiss TPH, Basel, Switzerland; University of Basel, Basel, Swtizerland); **Viktor von Wyl** (Epidemiology, Biostatistics and Prevention Institute, University of Zurich, Zurich, Switzerland); **Cornelia Wagner**, MSc (Population Health Laboratory (#PopHealthLab), University of Fribourg, Switzerland); **Rylana Wenger** (Institute of Primary Health Care (BIHAM), University of Bern, Department of General Internal Medicine, Inselspital, Bern University Hospital, University of Bern); **Erin West**, PhD (Epidemiology, Biostatistics and Prevention Institute, University of Zurich, Zurich, Switzerland); **Ania Wisniak**, MD (Unit of Population Epidemiology, Division of Primary Care Medicine, Geneva University Hospitals, Geneva, Switzerland; Institute of Global Health, Faculty of Medicine, University of Geneva, Geneva, Switzerland); **María-Eugenia Zaballa**, PhD (Unit of Population Epidemiology, Division of Primary Care Medicine, Geneva University Hospitals, Geneva, Switzerland); **Claire Zuppinger** (Center for Primary Care and Public Health (Unisanté), University of Lausanne, Switzerland)
