# Supplementary material for: The Corona Immunitas Digital Follow-Up eCohort to Monitor Impacts of the SARS-CoV-2 Pandemic in Switzerland: Study Protocol and First Results
Source: Int J Public Health. 2022 Feb 28;67:1604506. doi: 10.3389/ijph.2022.1604506 (PMC8919370; doi:10.3389/ijph.2022.1604506)
Supplement: Supplementary file 2 [file DataSheet1.PDF]

International Journal of Public Health

The Corona Immunitas Digital Follow-Up eCohort to monitor impacts of the SARS-CoV-2 pandemic in Switzerland: Study protocol and first results

Weekly and Monthly Questionnaires (September 2020-February 2021): Please contact the Corona Immunitas secretariat (hello@corona-immunitas.ch) for use.

WEEKLY FOLLOW-UP

Date *(today's)* ____________________________________

1 Over the past 7 days, have you had any new symptoms unrelated to pre-existing chronic illness or allergies? O Yes O No

→ If NO, please go directly to 2

1.1 During the last 7 days, have you had the following symptoms (new symptoms unrelated to pre-existing chronic illness or allergies?

→ Please select yes or no for each symptom.

	**Yes**	**No**
Feeling feverish	O	O
Body temperature of 38°C/100.4°F or more	O	O
Dry cough	O	O
Coughing with sputum	O	O
Bloody sputum	O	O
Runny or blocked nose	O	O
Sneezing	O	O
Sore throat	O	O
Shortness of breath	O	O
Breathing difficulties	O	O
Headaches	O	O
Muscle and/or limb pain	O	O
Pain in the chest, thorax and/or sternum	O	O
Tiredness or exhaustion	O	O
Loss of appetite	O	O
Nausea and/or vomiting	O	O
Diarrhea	O	O
Abdominal pain	O	O
Loss of smell and/or taste	O	O
Irritated and/or watery eyes	O	O
Other symptoms	O	O

If other symptoms*, please specify:* ____________________________________

1.2 When the first symptoms appeared, what did you do? (You can select multiple answers)

- You called your doctor;
- You went to the doctor's office;
- You went to a hospital emergency room;
- You called a coronavirus medico-sanitary hotline of the Service du médecin cantonal;
- You called emergency services (144);
- You called a medical counselling service (e.g. insurance) ;
- You went to the pharmacy;
- You stayed home;
- Other ________________________________

1.3 Over the past 7 days, have you taken any medicine to treat those new symptoms? (You may select multiple answers)

- No medication
- Paracetamol (e.g. Dafalgan, NeoCitran, Pretuval, Panadol, Ben-U-RON, Zolben, etc.)
- Cortisone, Prednisone
- Non-steroidal analgesics/anti-inflammatory drugs (e.g. Ibuprofen, Irfen, Algifor, Brufen, etc.)
- Other painkillers/inflammation inhibitors (e.g. Aspirin, Naproxen, Diclofenac, etc.)
- Antiviral agents (e.g. Tamiflu, Ritonavir-Lopinavir, Remdesivir, etc.)
- Antibiotics (e.g. Amoxicillin, Azithromycin, Bactrim, etc.)
- Nasal sprays for asthma or chronic bronchitis (e.g. Atrovent, Bricanyl, Dospir, Seretide, Ventolin, Symbicort, Spiriva, etc.)
- Hydroxychloroquin (Plaquentil)
- Other medicines (please specify):
- ________________________________

1.4 Over the past 7 days, have you been hospitalised because of the symptoms you're reporting today?

- Yes
- No

1.1.4 If yes, how long did the hospitalisation last? (Number of days)

-

Date of Start (dd/mm/yyyy) ________________________________

Date of End (dd/mm/yyyy) ________________________________

I'm still hospitalised O Yes O No

1.4.2 During this hospitalisation, have you been admitted to an intensive care unit?

If yes, how many days?

---

2. Over the past 7 days, have you been tested for coronavirus (SARS-CoV-2)?

- Yes

(nasal or oral swab)

- No
- I don’t know / I don’t want to answer this question

2.1 If yes, at what date? (dd/mm/yyyy) ____________________

2.2 If yes, the test result was

- Positive (Virus present)
- Negative (Virus not present)
- Waiting for results
- I don’t know / I don’t want to answer this question

2.3 Who conducted this test?

- Health care professional
- Self test
- Family member or friend

2.4 What type of test was it?

- PCR-Test (Test conducted in a Laboratory, received between 24-48 hours)
- Antigen-rapid test (Results in 15-30 minutes)
- Covid-Selftest (At home test)
- I don’t know

3. Over the past 7 days, have you been medically followed-up at home by a coronavirus medical follow-up (provided by your canton)?

O Yes O No

4. Over the past 7 days, have you done a blood test (serology) to detect if you had antibodies against SARS-CoV-2?(other than Corona-Immunitas study)

- Yes
- No

I don’t know / I don’t want to answer this question

4.1 If yes, at what date? (dd/mm/yyyy) ____________________

4.2 If yes, the test result was

- Positive (Antibodies present)
- Negative (Antibodies not present)
- Test result still pending

I don’t know / I don’t want to answer this question

In the last seven days, how many times have you …

	Never	Very rare	Occasionally	Frequently	Always
5. ... applied the recommended hygiene measures (washing hands regularly, sneezing into the elbow, using disposable tissues, etc.)?	O	O	O	O	O
6. ... applied social distancing measures (avoid shaking hands or	O	O	O	O	O

kissing, avoid hugging, staying at a distance of 2 metres)?					
7. ... applied the recommendations for staying at home (staying at home as much as possible, avoiding unnecessary outings or trips, etc.)?	O	O	O	O	O
8. ... worn a mask to protect yourself and others from coronavirus (SARS-CoV-2)?	O	O	O	O	O

Not at all ____ **Very high**

9. In the last 7 days, do you think the risk of being infected with COVID-19 is… *(Mark on the line where you believe the risk is; if you do not know, please check "I don't know")*

- I don't know

10. For the past 7 days, have you been forced to stay at home because of exposure to coronavirus (SARS-CoV-2)?

- Yes, because I have tested positive for coronavirus and/or have symptoms
- Yes, because I was in contact with a person who tested positive for coronavirus
- Yes, because a household member has tested positive for coronavirus or has symptoms
- Yes, because I received an alert from the SwissCovid App that I have a risk of contagion.
- Yes, because I am at risk (because of my age or a previous illness
- Yes, because I was more than 24 hours in an area, which according to the Federal Office has quarantine obligation for health reasons.
- Yes, for another reason
- No
- I don't know/don't want to answer

***If yes for another reason**, please specify:*

____

10.1 If you stayed at home (or are still at home), who asked you to do so

- I have followed the prescription of my doctor or the health specialist following me
- I have followed the health recommendations currently applied in my canton
- I have followed the recommendations of my employer or my occupational physician
- I followed the recommendations of my relatives (friends, family)
- I decided to stay home by myself
- Other

***If other**, please specify:*

____

MONTHLY FOLLOW-UP

Date *(today's)* ______________________

1 Over the past 7 days, have you had any new symptoms unrelated to pre-existing chronic illness or allergies? O Yes O No

→ If NO, please go directly to 2

1.1 During the last 7 days, have you had the following symptoms (new symptoms unrelated to pre-existing chronic illness or allergies?

→ Please select yes or no for each symptom.

	Yes	No
Feeling feverish	O	O
Body temperature of 38°C/100.4°F or more	O	O
Dry cough	O	O
Coughing with sputum	O	O
Bloody sputum	O	O
Runny or blocked nose	O	O
Sneezing	O	O
Sore throat	O	O
Shortness of breath	O	O
Breathing difficulties	O	O
Headaches	O	O
Muscle and/or limb pain	O	O
Pain in the chest, thorax and/or sternum	O	O

	Yes	No
Tiredness or exhaustion	O	O
Loss of appetite	O	O
Nausea and/or vomiting	O	O
Diarrhea	O	O
Abdominal pain	O	O
Loss of smell and/or taste	O	O
Irritated and/or watery eyes	O	O
Other symptoms	O	O

***If other symptoms**, please specify:* ______________________

1.2 When the first symptoms appeared, what did you do? (You can select multiple answers)

- You called your doctor;
- You went to the doctor's office;
- You went to a hospital emergency room;
- You called a coronavirus medico-sanitary hotline of the Service du médecin cantonal;
- You called emergency services (144);
- You called a medical counselling service (e.g. insurance) ;

- o You went to the pharmacy;
- o You stayed home;
- o Other ______________________________

1.3 Over the past 7 days, have you taken any medicine to treat those new symptoms? *(Multiple answers possible)*

- o No medication
- o Paracetamol (e.g. Dafalgan, NeoCitran, Pretuval, Panadol, Ben-U-RON, Zolben, etc.)
- o Cortisone, Prednisone
- o Non-steroidal analgesics/anti-inflammatory drugs (e.g. Ibuprofen, Irfen, Algifor, Brufen, etc.)
- o Other painkillers/inflammation inhibitors (e.g. Aspirin, Naproxen, Diclofenac, etc.)
- o Antiviral agents (e.g. Tamiflu, Ritonavir-Lopinavir, Remdesivir, etc.)
- o Antibiotics (e.g. Amoxicillin, Azithromycin, Bactrim, etc.)
- o Nasal sprays for asthma or chronic bronchitis (e.g. Atrovent, Bricanyl, Dospir, Seretide, Ventolin, Symbicort, Spiriva, etc.)
- o Hydroxychloroquin (Plaquentil)
- o Other medicines (please specify):

____

1.4 Over the past 7 days, have you been hospitalised because of the symptoms you're reporting today?

o Yes o No

1.1.4 If yes, how long did the hospitalisation last? (Number of days)

o

Date of Start (dd/mm/yyyy) ______________________________

Date of End (dd/mm/yyyy) ______________________________

I'm still hospitalised O Yes O No

1.4.2 During this hospitalisation, have you been admitted to an intensive care unit?

If yes, how many days?

---

2. Over the past 7 days, have you been tested for coronavirus (SARS-CoV-2)? (nasal or oral swab)

- o Yes
- o No
- o I don't know / I don't want to answer this question

2.1 If yes, at what date? (dd/mm/yyyy)

____

2.2 If yes, the test result was

- o Positive (Virus present)
- o Negative (Virus not present)
- o Waiting for results
- o I don't know / I don't want to answer this question

2.3 Who conducted this test?

- o Health care professional

- o Self test
- o Family member or friend

2.4 What type of test was it?

- o PCR-Test (Test conducted in a Laboratory, received between 24-48 hours)
- o Antigen-rapid test (Results in 15-30 minutes)
- o Covid-Selftest (At home test)
- o I don't know

3. Over the past 7 days, have you been medically followed-up at home by a coronavirus medical follow-up (provided by your canton)?

O Yes O No

4. Over the past 7 days, have you done a blood test (serology) to detect if you had antibodies against SARS-CoV-2?(other than Corona-Immunitas study)

- o Yes
- o No

I don't know / I don't want to answer this question

4.1 If yes, at what date? (dd/mm/yyyy)

____

4.2 If yes, the test result was

- o Positive (Antibodies present)
- o Negative (Antibodies not present)
- o Test result still pending

I don't know / I don't want to answer this question

4.1 In the last seven days, how many times have you …

	**Never**	**Very rare**	**Occasionally**	**Frequently**	**Always**
… implemented the recommended measures for "**social distancing"** (no handshaking or hugging, keep the distance of 2 meters, etc.)?	O	O	O	O	O
… implemented the recommendation to **stay at home** (stay at home whenever possible, avoid unnecessary activities outside the home, etc.)?	O	O	O	O	O
… worn a **mask** to protect yourself and others from the coronavirus (SARS-CoV-2)?	O	O	O	O	O
… implemented the recommended **hygiene measures** (washing hands regularly, sneezing into the elbow, using tissues, etc.)?	O	O	O	O	O

4.2 In the last 7 days, do you think the risk of being infected with COVID-19 is… *(Mark on the line where you believe the risk is; if you do not know, please check "I don't know")*

Not at all ________________________________ **Very high**

- o I don't know

4.3 For the past 7 days, have you been forced to stay at home because of exposure to coronavirus (SARS-CoV-2)?

- Yes, because I have tested positive for coronavirus and/or have symptoms
- Yes, because I was in contact with a person who tested positive for coronavirus
- Yes, because a household member has tested positive for coronavirus or has symptoms
- Yes, because I am at risk (because of my age or a previous illness
- Yes, for another reason
- No
- I don't know/don't want to answer

***If yes for another reason**, please specify:* ______________________________

***In the last seven days**, how many times have you …*

	Never	Very rare	Occasionally	Frequently	Always
5. ... applied the recommended hygiene measures (washing hands regularly, sneezing into the elbow, using disposable tissues, etc.)?	O	O	O	O	O
6. ... applied social distancing measures (avoid shaking hands or kissing, avoid hugging, staying at a distance of 2 metres)?	O	O	O	O	O
7. ... applied the recommendations for staying at home (staying at home as much as possible, avoiding unnecessary outings or trips, etc.)?	O	O	O	O	O
8. ... worn a mask to protect yourself and others from coronavirus (SARS-CoV-2)?	O	O	O	O	O

Not at all ______________________________ **Very high**

9. In the last 7 days, do you think the risk of being infected with COVID-19 is… *(Mark on the line where you believe the risk is; if you do not know, please check "I don't know")*

- I don't know

10. For the past 7 days, have you been forced to stay at home because of exposure to coronavirus (SARS-CoV-2)?

- Yes, because I have tested positive for coronavirus and/or have symptoms
- Yes, because I was in contact with a person who tested positive for coronavirus
- Yes, because a household member has tested positive for coronavirus or has symptoms

- o Yes, because I received an alert from the SwissCovid App that I have a risk of contagion.
- o Yes, because I am at risk (because of my age or a previous illness
- o Yes, because I was more than 24 hours in an area, which according to the Federal Office has quarantine obligation for health reasons.
- o Yes, for another reason
- o No
- o I don't know/don't want to answer

If yes for another reason*, please specify:* ____

10.1 If you stayed at home (or are still at home), who asked you to do so

- o I have followed the prescription of my doctor or the health specialist following me
- o I have followed the health recommendations currently applied in my canton
- o I have followed the recommendations of my employer or my occupational physician
- o I followed the recommendations of my relatives (friends, family)
- o I decided to stay home by myself
- o Other

If other*, please specify:* ____

11. How worried are you about the current coronavirus situation in the following areas:

	**Not at all**	**A little**	**Moderate**	**Very**	**Extreme**
The consequences for my health	O	O	O	O	O
The health of relatives and friends	O	O	O	O	O
The risk of exposing myself to the virus	O	O	O	O	O
The risk of spreading the virus	O	O	O	O	O
My own economic and/or professional situation	O	O	O	O	O
The economic and/or professional situation of relatives and friends	O	O	O	O	O
The general economic situation in Switzerland	O	O	O	O	O
The quality of my family relationships	O	O	O	O	O
The quality of my personal relationships (e.g. with friends and colleagues)	O	O	O	O	O

The freedom of Swiss citizens	O	O	O	O	O
The privacy of Swiss citizens	O	O	O	O	O

12. To which extent do you think the following people are worried about the current coronavirus (SARS-CoV-2) situation:

	**Not at all**	**A little**	**Moderate**	**Very**	**Extreme**
My family members	O	O	O	O	O
Other people around me (e.g. friends, neighbors, colleagues)	O	O	O	O	O
Public health authorities	O	O	O	O	O
News media	O	O	O	O	O
People on social media	O	O	O	O	O
Healthcare practitioners	O	O	O	O	O
Politicians	O	O	O	O	O

13. During the past 7 days, how often did you feel that you lacked companionship)?

- Never
- Rarely
- Occasionally
- Most of the time
- Always

14. During the past 7 days, how often did you feel left out?

- Never
- Rarely
- Occasionally
- Most of the time
- Always

15. During the past 7 days, how often did you feel isolated from others?

- Never
- Rarely
- Occasionally
- Most of the time
- Always

17. Did you need medical treatment because of an ongoing illness?

18. Have you changed your ongoing medical treatment during the past month?

- No, I have not changed my medical treatment
- Yes, I have had problems obtaining my usual treatment
- Yes, I have stopped my treatment (for example: corticosteroids, anti-inflammatory drugs) so as not to risk aggravating a potential coronavirus infection
- Yes, for another reason

18.1 Have you been afraid of getting infected with the coronavirus (SARS-CoV-2) by going

- Yes, very afraid
- Yes, fairly afraid

for treatment?

- o No, not very afraid
- o No, not afraid of everything
- o Not concerned

18.2 Why did you have problems obtaining your usual treatment *(select all that apply)****?***

- o My appointment was postponed or cancelled
- o The health professional had closed his/her office
- o I couldn't have my children looked after to go for treatment
- o Other reason

Please specify: ___________________________

18.3 Why have you stopped your treatment *(select all that apply)****?***

- o Because I was too afraid of being infected during treatment
- o Because I wanted to reduce my trips outside the home
- o Because I wanted to protect other members in my family from getting infected
- o Other reason
- o *Please specify:* ___________________________

If yes for another reason*, please specify:* ____

19. During the past month, did you need to see a health professional for an acute health problem not related to the Coronavirus (SARS-CoV-2) and not part of an ongoing treatment?

O Yes O No

19.1 If yes, how did this consultation take place?

- o By phone or telemedicine
- o At home
- o In a doctor's office
- o In a hospital department
- o In hospital emergency rooms
- o Other

19.1.1. Have you been afraid of being infected with the Coronavirus (SARS-CoV-2) during the consultation?

O Yes O No

If yes for another reason*, please specify:* ____

The SwissCovid App has been launched by the Swiss Federal Office of Public Health to warn smartphone users in case of possible exposure risks. The app records if a contact has been in close proximity of 1.5m or less for longer than 15 minutes. If an app user tested positive for the coronavirus, she or he can anonymously notify other app users who were in close proximity during the infectious period.

Are you using the SwissCovid App?

- o Yes, permanently
- o Yes, but sometimes I turn off Bluetooth to pause the SwissCovid app
- o No, but I'm planning to use it
- o No

Were you ever notified by the SwissCovid App that you have been in close proximity to a corona-positive person)?

- o No, I have never received a notification
- o Yes, I called the recommended info line for SwissCovid

	o Yes, I undertook other steps<br>*Please specify:* ___________________________<br>o Yes, but I did not undertake any steps
Why are you currently not using the SwissCovid App?	o I have not heard about the app<br>o I don't think the app is useful for me<br>o I can't install the app (e.g. owing to technical difficulties or because I do not own an Android or iOS smartphone)<br>o I fear for my privacy and protection of my data<br>o Other reasons<br>o *Please specify:* ___________________________

What motivates you to be a part of Corona Immunitas? What is your experience with our study?

____

____

QUESTIONNAIRES NOT AVAILABLE EVERY MONTH:

Please read each statement and indicate a number 0, 1, 2, or 3, which indicates how much the statement applied to you over the past week. There are no right or wrong answers. Do not spend too much time on any statement:

	**0 – Did not apply to me at all**	**1 – Applied to me to some degree, or some of the time**	**2 – Applied to me to a considerable degree, or a good part of the time**	**3 – Applied to me very much, or most of the time**
I found it hard to wind down	O	O	O	O
I was aware of dryness of my mouth	O	O	O	O
I couldn't seem to experience any positive feelings at all	O	O	O	O
I experienced breathing difficulty (e.g. excessively rapid breathing, breathlessness in the absence of physical exertion)	O	O	O	O
I found it difficult to work up the initiative to do things	O	O	O	O
I tended to over-react to situations	O	O	O	O
I experienced trembling (e.g. in the hands)	O	O	O	O
I felt that I was using a lot of nervous energy	O	O	O	O
I was worried about situations in which I might panic and make a fool of myself	O	O	O	O
I felt that I had nothing to look forward to	O	O	O	O

Please read each statement and indicate a number 0, 1, 2, or 3, which indicates how much the statement applied to you over the past week. There are no right or wrong answers. Do not spend too much time on any statement:

	**0 – Did not apply to me at all**	**1 – Applied to me to some degree, or some of the time**	**2 – Applied to me to a considerable degree, or a good part of the time**	**3 – Applied to me very much, or most of the time**
I found myself getting agitated	O	O	O	O
I found it difficult to relax	O	O	O	O
I felt down-hearted and blue	O	O	O	O
I was intolerant of anything that kept me from getting on with what I was doing	O	O	O	O
I felt I was close to panic	O	O	O	O
I was unable to become enthusiastic about anything	O	O	O	O
I felt I wasn't worth much as a person	O	O	O	O
I felt that I was rather touchy	O	O	O	O
I was aware of the action of my heart in the absence of physical exertion (e.g. sense of heart rate increase, heart missing a beat)	O	O	O	O
I felt scared without any good reason	O	O	O	O
I felt that life was meaningless	O	O	O	O
