# Supplementary material for: The Corona Immunitas Digital Follow-Up eCohort to Monitor Impacts of the SARS-CoV-2 Pandemic in Switzerland: Study Protocol and First Results
Source: Int J Public Health. 2022 Feb 28;67:1604506. doi: 10.3389/ijph.2022.1604506 (PMC8919370; doi:10.3389/ijph.2022.1604506)
Supplement: Supplementary file 3 [file DataSheet2.DOCX]

International Journal of Public Health

**The Corona Immunitas Digital Follow-Up eCohort to monitor impacts of the SARS-CoV-2 pandemic in Switzerland: Study protocol and first results**

Supplementary materials

**Supplementary Figure 1: Cumulative enrolment and participation rate for all five sites (Zurich, Basel, Fribourg, Neuchatel, and Ticino).**


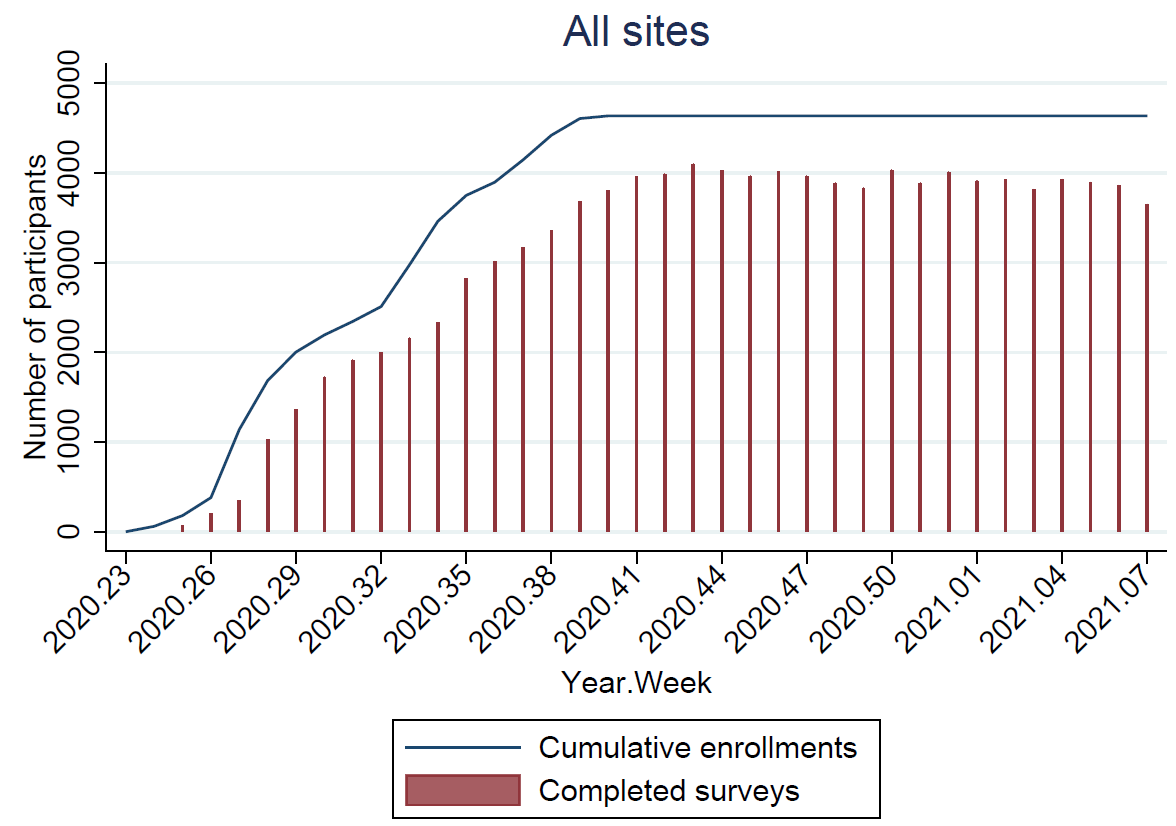


**Supplementary table 1: CI-DFU Operational differences across sites**

|  | **Basel (City/Land)** | **Bern** | **Fribourg** | **Geneva** | **Grisons** | **Lausanne** | **Lucerne** | **Neuchatel** | **St. Gallen** | **Ticino** | **Zurich** |
| --- | --- | --- | --- | --- | --- | --- | --- | --- | --- | --- | --- |
| Study Enrolment/Study population | Random sample of both cantonal populations^1^ + household members (including children aged 7 years and older) | Random sample of the cantonal population^1^ | Random sample of the cantonal population^1^ | Phase 1-2 : Participants of a previous study (Bus Santé) with population-based sample and their household members  Phases 3-4: Random sample of the cantonal population^1^ and their household members if selected participant is a minor, + random selection of old participants of phase 1-2 | Random sample of the cantonal population^1^ + householders (one additional participant from the same household) | Random sample of the cantonal population^1^ | Random sample of the cantonal population^1^ | Random sample of the cantonal population^1^ | Random sample of the cantonal population^1^ + householders (one additional participant from the same household) | Random sample of the cantonal population^1^ (FSO) + householders (HH) (grandparents/grandchildren) | Random sample of the cantonal population^1^ |
| Frequency of sero-prevalence follow-up | One time at baseline for Seroprevalence Cohort arm AND twice (at least 2 months later) for all households and all serologically positive subjects;  None for Digital cohort arm. | One time at baseline | One time at baseline | Approx. every 6 months for a subsample of the population | One time at baseline | One time at baseline | One time at baseline | One time at baseline | One time at baseline | Subgroup 20-64: 2 FUs, after 4 and 12 months.  Subgroups 5-19 and 65+: 1 FU after approx. 6 months | Nested control subgroup: 149 individuals 3 FUs, after 4, 6, 10 month  Repeated test in phase 3: 493 individuals |
| Data Collection Scheduling | Weekly and monthly on a calendar-based sequence | Weekly and monthly on a calendar-based sequence | Weekly and monthly on a calendar-based sequence | Weekly and monthly on a calendar-based sequence + unique thematic questionnaires (i.e. vaccination, mental health … ) | Weekly and monthly on a calendar-based sequence | Weekly and monthly on a calendar-based sequence | Weekly and monthly on a calendar-based sequence | Weekly and monthly on a calendar-based sequence | Weekly and monthly on a calendar-based sequence | Weekly and monthly on a sequential study follow-up (the Baseline questionnaire completion date determines the delivery of questionnaire) | Weekly and monthly on a calendar-based sequence |
| Data Collection tools | REDCap | REDCap | REDCap | ww.specchio-covid19.ch (SugarCRM-based solution) | REDCap | REDCap |  | REDCap | REDCap | REDCap | REDCap |

*Note*: ^1^ provided by the Swiss Federal Statistical Office

**Supplementary Table 2a. CI-DFU enrolment in Phase 2**

|  | **Basel (City/Land)** | **Fribourg** | **Neuchâtel** | **Ticino*** | **Zurich** |
| --- | --- | --- | --- | --- | --- |
| Start Date (Phase 2) | 11.07.2020 | 24.06.2020 | 14.08.2020 | 01.07.2020 | 11.6.2020 |
| Age-subgroups | 7-17; 18-49; 50-64; 65+ | 20-64, 65+ years | 20-64, 65+ years | 20-64 | 20-64, 65+ years |
| Number giving informed consent for CI-DFU | 2269 | 308 | 284 | 978 | 797 |
| Number (percent of eligible) having completed at least one digital follow-up | 2106 (92.8) | 231 (75) | 230 (81.0) | 833(85.2) | 706 (88.6) |

* By design, participants were invited to the CI-DFU first, and a sub-sample was then invited to take part in the seroprevalence part of the parent CI-study.

**Supplementary table 2b: CI-DFU enrolment in Phase 3**

|  | **Basel (City/Land)** | **Bern** | **Fribourg** | **Geneva** | **Grison** | **Lucerne** | **Neuchâtel** | **St. Gallen** | **Ticino*** | **Vaud** | **Zurich** |
| --- | --- | --- | --- | --- | --- | --- | --- | --- | --- | --- | --- |
| Start Date (Phase 3) | 13.01.2021 | 14.12.2020 | 04.11.2020 | 23.11.2020 | 03.02.2021 | 25.01.2021 | 23.12.2020 | 02.12.2020 | 14.11.2020 | 18.01.2021 | 24.11.2020 |
| Age-subgroups | 7-17; 18-49; 50-64; 65+ | 20-64, 65+ years | 20-64, 65+ years | 18-64, 65+ years | 5-13; 14-19; 20-64 | 18-64, 65+ years | 20-64, 65+ years | 5-13; 14-19; 20-64 | 5-13; 14-19; 65+ years | 15-64, 65+ years | 20-64,  65+ years |
| Number giving informed consent for CI-DFU | 902 | 338 | 541 | 7647 | 339 | 424 | 422 | 303 | 5-13: 629  14-19: 451  65+: 882 | 1189 | 585 |

* By design, participants were invited to the CI-DFU first, and a sub-sample was then invited to take part in the seroprevalence part of the parent CI-study.
